# Rapid Progression of Aortitis Caused by Methicillin-Sensitive Staphylococcus Aureus in a Patient With Pneumonia: A Case Report

**DOI:** 10.7759/cureus.54674

**Published:** 2024-02-22

**Authors:** Hideya Itagaki, Tomoya Ooizumi, Chiho Sanada, Yoshinobu Abe, Tomoyuki Endo

**Affiliations:** 1 Department of Emergency and Disaster Medicine, Tohoku Medical and Pharmaceutical University Hospital, Sendai, JPN; 2 Department of Emergency Medicine, South Miyagi Medical Center, Sendai, JPN

**Keywords:** staphylococcus aureus, rupture, infected aortic aneurysm, pleurisy, pneumonia

## Abstract

Infected aortic aneurysm is a rare but fatal disease that occurs through various mechanisms. In this report, we describe the case of a patient who was hospitalized for acute pneumonia and developed an infected aortic aneurysm in the descending aorta during the hospitalization. A 73-year-old Japanese man presented to the emergency department with a chief complaint of fever. He had a history of chronic renal failure due to nephrosclerosis and was on regular hemodialysis three times a week. The patient presented with an elevated inflammatory response, anemia, and low platelet counts after various tests. Computed tomography (CT) showed ground-glass opacity in the left lung with a small amount of pleural effusion, leading to a diagnosis of pneumonia. The patient was admitted to the hospital on the same day, and a course of antibiotics (ceftriaxone [CTRX]) was started. On the fourth day of hospitalization, *methicillin-susceptible Staphylococcus aureus* (MSSA) was detected in the blood sample, which was collected from the patient on the day of admission. The patient was treated for MSSA pneumonia and bacteremia, and the antibiotics were changed to cefazolin (CEZ). Treatment with antimicrobials resulted in a negative blood culture retest on day 5 and improvement of the inflammatory response. On the 12th day, improvements in pneumonia and pleurisy were observed on the CT scan; however, an abnormal bulge was seen on the dorsal side of the descending thoracic aorta with suspected partial vessel wall disruption, suggesting a ruptured infected aortic aneurysm. Despite treatment with antibiotics, the thoracic descending aortic aneurysm continued to dilate with progressing rupture, and the patient died on the 25th day of hospitalization.

This is the first report of an infected aneurysm caused by Staphylococcus aureus, despite a negative blood culture. Patients at high risk might develop infected aneurysms, and the possibility of rapid dilation should always be considered.

## Introduction

Aortitis can be caused by bacteria or fungi infection of the aortic wall [1]. Although infected aortic aneurysms are rare and account for less than 2% of all aortic aneurysms, these can be fatal despite various advancements in medicine [2]. Infected aneurysms are treated by surgery and with long-term antibiotics. However, it is essential to note that they can dilate rapidly and rupture if left untreated, indicating the need for prompt diagnosis and treatment.

Herein, we report the case of an elderly male with pneumonia who developed an infected aneurysm post-hospitalization, which rapidly expanded and ruptured because of an infection in the descending thoracic aorta adjacent to the lesion, despite showing improvements in the chest examination and blood culture tests.

## Case presentation

A 73-year-old Japanese man presented to the emergency department with a chief complaint of fever. He was on intermittent hemodialysis three times a week for chronic renal failure due to nephrosclerosis. After regular hemodialysis, the patient developed a fever (39°C) with chills. He was taken to the emergency room. He had no fever on the day before the visit. Other medical history included hypertension, gastric ulcer, angina pectoris, and arteriosclerosis obliterans, and he was taking the following medications for his conditions: cilostazol, clopidogrel, bisoprolol, lafutidine, and amlodipine. His family history was unremarkable, and he did not smoke or drink alcohol. During the emergency room visit, the vital signs were blood pressure 171/70 mmHg, pulse rate 86 times/minute, respiratory rate 26 times/minute, body temperature 40.5°C, and SpO_2_ 96%. Blood tests, blood culture tests, and computed tomography (CT) scanning were performed to identify the source of the fever. Blood tests revealed elevated inflammatory makers, including white blood cell count (WBC), 10,600 cell/μL; C-reactive protein (CRP), 14.84 mg/dL; procalcitonin, 5.56 ng/mL; anemia (hemoglobin, 9.2 g/dL), and a low platelet count (87,000/μL), whereas the results of the coagulation tests (prothrombin time, 13.7 s, and activated partial thromboplastin time, 38.4 s) were normal (Table 1).

**Table 1:** Post-hospitalization blood test results Tbil: Total bilirubin; AST: Aspartate aminotransferase; ALT: Alanine aminotransferase; LDH: Lactate dehydrogenase; BUN: Blood urea nitrogen; Cre: Creatinine; CRP: C-reactive protein; APTT: Activated partial thromboplastin time.

		Admission	Day 2	Day 5	Day 10	Day 12	Day 14	Day 17	Day 19	Day 22	Day 24
Tbil	mg/d	0.64	0.34	0.27	0.2	0.19	0.17	0.12	0.14	0.24	0.19
AST	U/L	22	40	18	13	12	9	8	8	8	6
ALT	U/L	13	17	9	<4	<4	<4	<4	<4	<4	<4
LDH	U/L	296	268	234	258	234	213	220	197	197	185
BUN	mg/dL	21	32	46	69	51	47	70	53	28	65
Cre	mg/dL	4.21	6.08	7.89	10.12	8.74	7.92	8.86	7.63	5.22	9.17
Na	mmol/L	138	137	133	133	137	137	133	131	134	131
K	mmol/L	3.9	3.8	3.9	5.5	5.1	5.1	5.6	5.6	5.2	6.6
CRP	mg/dL	14.84	20.21	24	13.57	10.82	6.66	7.21	5.52	9.62	14.47
Procalcitonin	ng/mL	5.56	18.12	18	-	-	2.11	1.69	1.61	1.67	2.7
WBC	10^3^/μL	10.6	10.6	9.3	12.1	12.3	12	9.2	7.8	7.7	7.8
RBC	10^6^/μL	2.82	2.74	2.75	2.58	2.56	2.42	2.19	2.22	2.56	2.24
Hb	g/dL	9.2	9	8.9	8.1	8	7.7	7	7	8.1	7.2
Ht	%	27.4	27.1	26.9	25.1	25	23.8	21.4	21.8	25.4	22.2
Plat	10^3^/μL	87	86	132	279	306	28.9	222	213	221	181
APTT	sec	38.4	-	43.1	-	42.4	39.2	42.5	41	48.4	49.5
PT	sec	13.7	-	14.4	-	14.4	14.2	13.9	13.8	14.1	14.6
D-dimer	μg/mL	5.19	-	5.97	-	4.28	4.33	3.31	2.87	2.49	4.05

**Figure 1:**
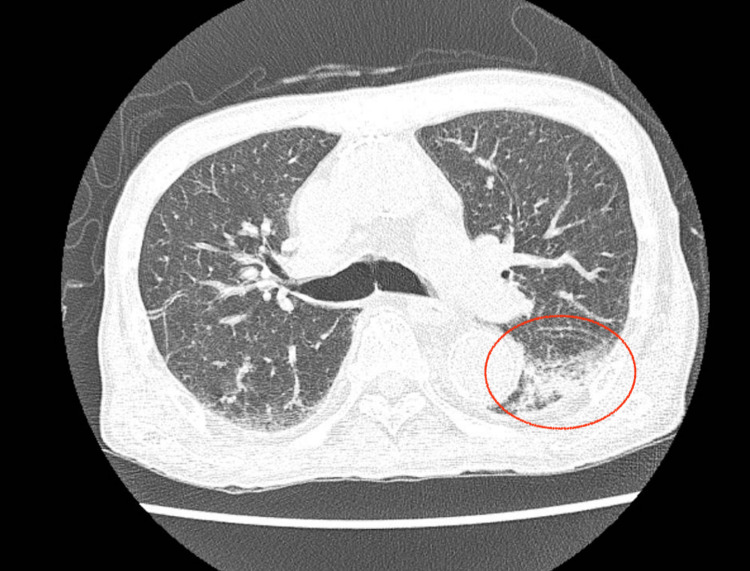
CT findings showing ground-glass opacity in the left lung (red circle)

The CT scans showed ground-glass opacity in the left lung with some pleural effusion (Figure 1).

**Figure 2:**
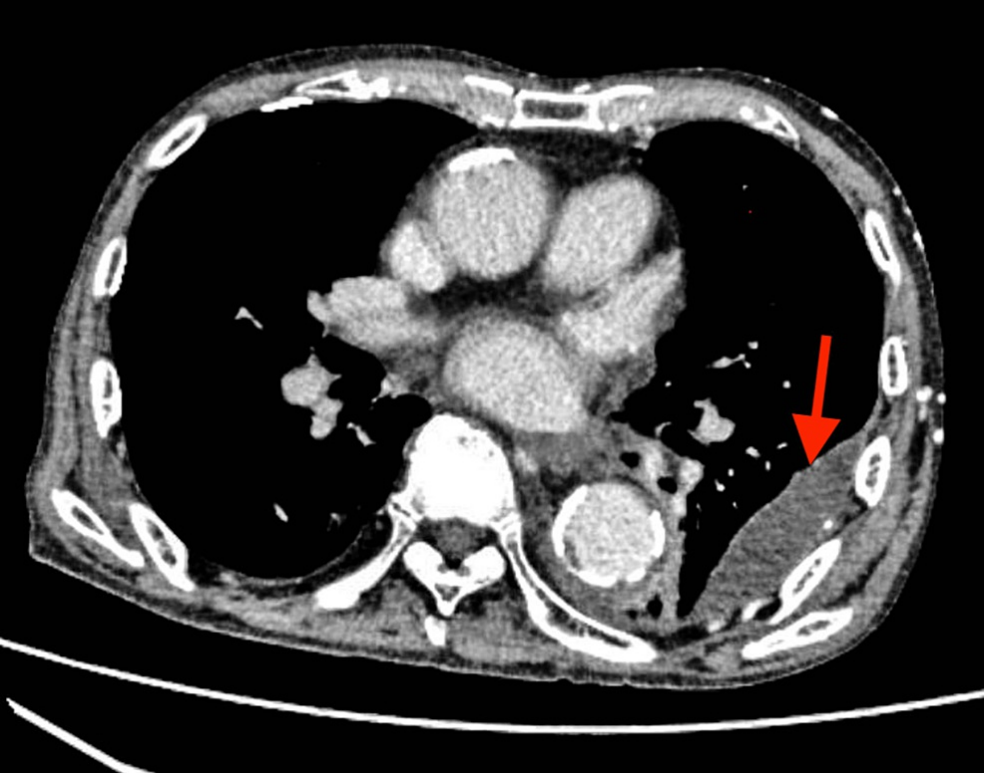
CT findings showing increased pleural effusion and pleurisy (red arrow)

There were no upper respiratory tract symptoms such as cough, but based on these results, pneumonia in the left lung was diagnosed. The patient was admitted to the hospital on the same day and started with a course of antibiotics, intravenous ceftriaxone (CTRX, 1 g/day). On the fourth day of hospitalization, two sets of blood cultures collected on admission were reported to be positive for methicillin-sensitive Staphylococcus aureus (MSSA) in both sets. Because of the poor quality of the sputum collected on the day of admission, only oral commensals were detected in the cultures. The patient was treated for MSSA pneumonia and bacteremia, and the antibiotics were changed to intravenous cefazolin (CEZ, 2 g/day + 1 g/day of dialysis). On the same day, the patient developed left lower back pain, and a CT scan revealed a slight increase in left pleural effusion and pleurisy (Figure 2).

**Figure 3:**
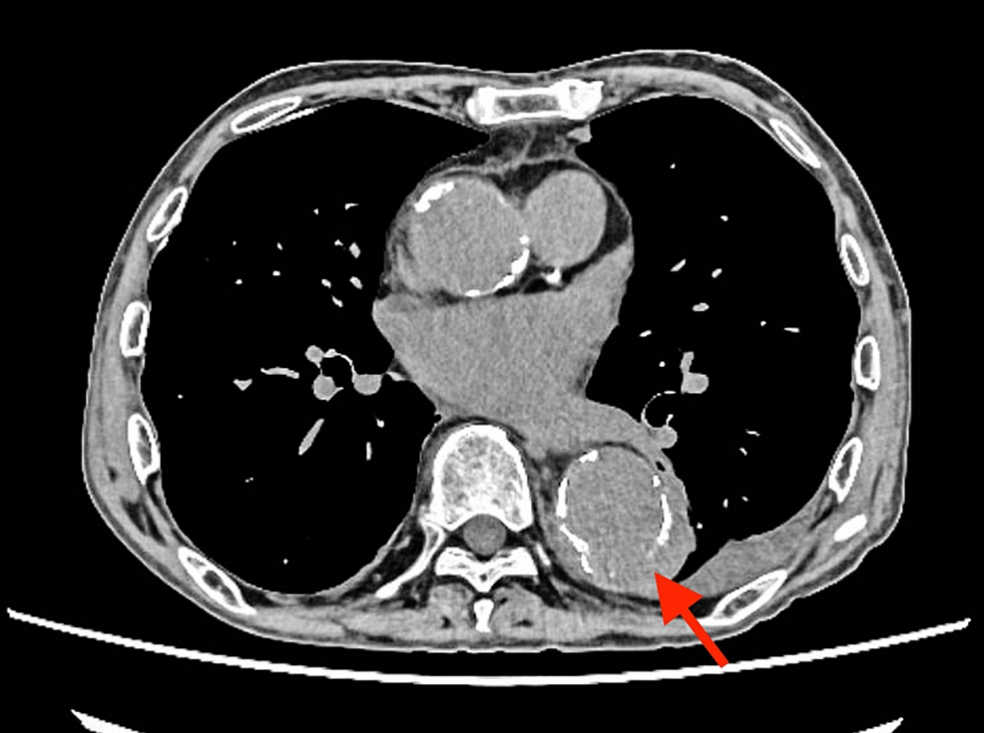
CT findings showing thoracic aorta with suspected partial vessel wall disruption (red arrow)

On the seventh day of hospitalization, a second blood culture retested on the fifth day was negative, and the leukocyte counts and CRP levels indicated a gradually improved inflammatory response. On the 12th day, another CT scan revealed improvements in pneumonia and pleurisy; however, a protruding aneurysm-like structure was observed on the dorsal side of the descending thoracic aorta with suspected partial vessel wall disruption, suggesting a ruptured infected aortic aneurysm (Figure 3).

**Figure 4:**
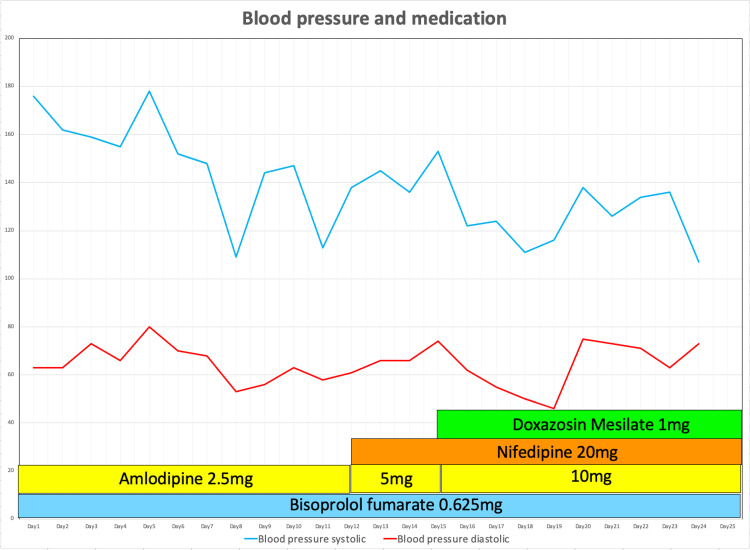
Blood pressure and medication

We consulted a vascular surgeon for treatment, and the vascular surgeon considered the replacement of the blood vessel with an artificial heart-lung machine or stent graft intubation. Stent graft intubation was difficult because of the small diameter of the patient's blood vessels. Replacement of the blood vessel was not indicated because of the high burden on the patient and the high risk of stroke. So, the vascular surgeon decided that surgery would be difficult. Hence, it was decided to continue with antibiotic therapy and control the blood pressure with antihypertensive agents. Subsequently, the blood pressure was controlled, and improved WBC and CRP levels were gradually observed in the blood tests (Figure 4).

**Figure 5:**
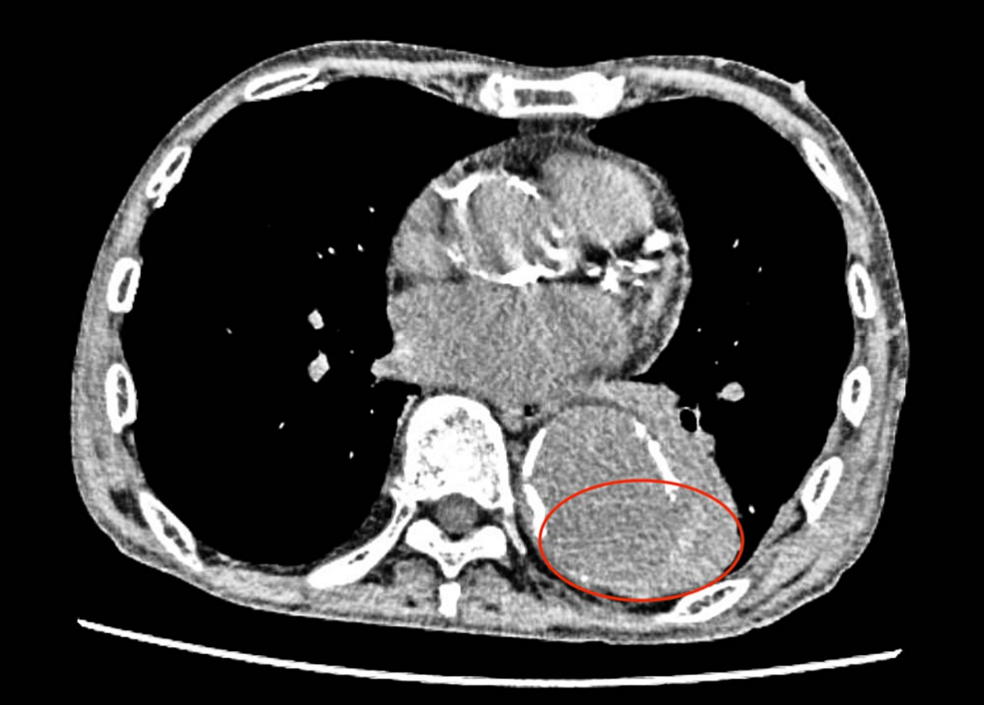
CT findings showing thoracic aortic aneurysm with irregular margins and partial high density (red circle)

On the 22nd day of hospitalization, the patient was eating less, complaining of severe back pain, and blood tests showed a re-elevated CRP. So, another CT scan was performed the next day (23rd day). The CT scan findings showed further dilatation of the aneurysm in the descending thoracic aorta with irregular margins, partial high absorption, and a progressive rupture (Figure 5).

**Figure 6:**
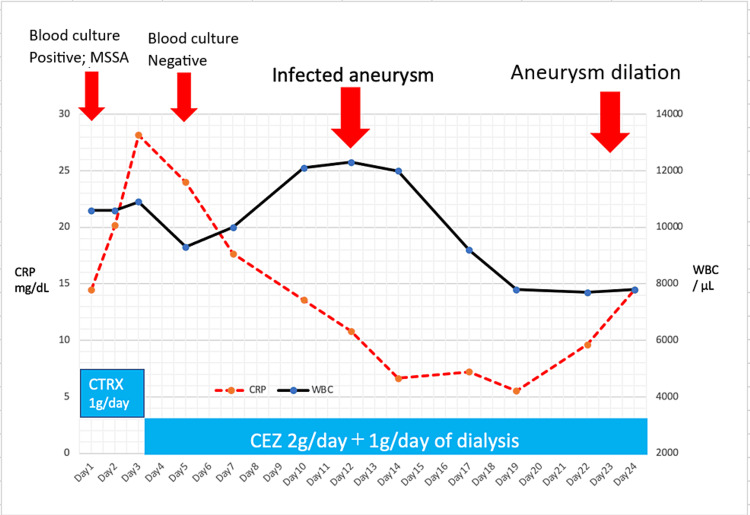
Systemic inflammation and the development of the aneurysm CTRX: Ceftriaxone; CEZ: Cefazolin.

After explaining the imaging results to the family, we decided to provide palliative care to reduce the back pain and started intravenous morphine. On the 24th day of hospitalization, the patient tended to be drowsy. The patient’s blood pressure subsequently decreased, and he died on the 25th day (Figure 6).

## Discussion

In this case, there are two points to consider. First, the pneumonia turned into an infectious aneurysm. Second, although the antibiotics used (CTRX and CEZ) were sensitive to MSSA, blood tests showed improvement in systemic inflammatory findings such as WBC and CRP, and reconstructed blood culture tests were negative; the infected aneurysm was completed in a short period of about one week.

Cases of pneumonia leading to infectious aneurysms are rare; only a few have been reported thus far [3,4]. The pathogenesis of an infected aneurysm, also called mycotic aneurysm [5], may be classified into four types: bacterial infiltration of the normal or abnormal intimal surface (atherosclerotic plaque or aneurysm), septic embolus occluding the vessel and causing infection, direct entry into the vessel from an infected site outside the vessel, and external implantation of contaminants into the vessel wall [6]. Normal arterial walls are resistant to bacterial invasion; however, patients with diabetes, alcoholism, cancer, dialysis, liver cirrhosis, malnutrition, and acquired immunodeficiency are susceptible to the infection [1,7]. The average age of patients susceptible to this condition is 65 years, which is more common in males [8]. The arteries most likely to be infected are the femoral artery, aorta, superior mesenteric artery, hepatic artery, splenic artery, and cerebral artery [8]. Various bacteria and fungi cause it, and the most common causative organisms are *Salmonella *and *Staphylococcus aureus*,* *which account for 28% of the infected aortic aneurysms [8].

In the present case study, the bacteria might have invaded the hardened arterial wall through the bloodstream or directly into the blood vessels from the pneumonia-affected areas and the associated pleural effusion in the left lung. This might be due to the calcification of the arterial wall as observed on the first CT scan, the reduced resistance to bacterial invasion, and the location of pneumonia and associated pleural effusion in contact with the aorta (possibility of direct invasion). However, the infection was not confirmed because the pleural fluid was not examined; nonetheless, a rapid, short-term increase in unilateral pleural effusions was strongly suspected of having been a complicated rather than simple pleural effusion, and the effusions could have been the cause of the problem. Additionally, the patient had a history of dialysis and was immunologically susceptible to infection. Furthermore, *S. aureus*, which is a common causative organism of infected aneurysms, was detected in the blood culture. Taken together, these findings indicate that the patient was susceptible to an infected aortic aneurysm.

Next, we would like to discuss how the infectious aneurysm developed in a short period, about one week, although the antibiotic was sensitive to MSSA and the retested blood culture was negative (Figure 6). Table 2 summarizes previous cases of infected aortic aneurysms, showing how long they have dilated or ruptured after infection [3,9-14]. Although it is commonly believed that infected aneurysms dilate rapidly, only a few reports on this phenomenon are found in the literature [10]. Rossi et al. reported a dilatation from 3.3 to 12 cm over an observation period of 50 days [11]. Similarly, Williams et al. reported a small dilatation (3.3-5 cm) over 10 days, whereas Ito et al. reported a 2.7-7 cm dilatation over seven days of observation [10,13]. Because of the variations in causative organisms involved in these studies, it is difficult to consider the causative organism as a factor leading to the expansion rate. However, in one study involving *S. aureus*, the aneurysm was dilated from 5.1 to 8 cm over an observation period of approximately three months [12]. In the current report, the aneurysm had dilated from 2.9 cm (Figure 2) to 3.6 cm (Figure 3) within one week and finally to 5.1 cm in three weeks (Figure 5), indicating the rapidity in the dilatation process.

**Table 2:** Previous cases of infected aortic aneurysms showing how long they have dilated or ruptured after infection

Author	Time for dilation (days)	Diameter (cm)	Pathogen	Antibiotics used	Blood culture (1st/ 2nd)	Course of inflammation
Williams et al. [10]	10	3.3→5	Streptococcus	Ampicillin and gentamicin	Not stated	WBC 11600→13600
Rossi and Cariati [11]	50	3.3→12	Streptococcus	Vancomycin, and piperacillin/tazobactam	Not stated	Not stated
Reslan et al. [12]	90	5.1→8	Staphylococcus aureus	Piperacillin/tazobactam, vancomycin, and metronidazole	Positive/No retested	WBC 50100
Ito and Takegoshi [13]	7	2.7→7	Klebsiella pneumoniae	Used (no name mentioned)	Positive/No retested	WBC 16000→Improvement: No values given
Furuta et al. [14]	10	Not stated	Escherichia coli	Cefepim and clindamycin	Positive/No retested	WBC 15800→12700
Zeng et al. [9]	40	Not stated	Salmonella	Ciprofloxacin	Negative	Not stated
Berdat et al. [3]	Several weeks	Not stated	Streptococcus pneumoniae	None	Not stated	Not stated

All patients with rapidly dilating infected aortic aneurysms in the studies enlisted in Table 2 were treated with antibiotics, except those with rupture. Despite antibiotic treatment, all the aortic aneurysms were dilated and required surgical intervention. Moreover, the dilatations were observed in patients with negative cultures and improved WBC levels following antibiotic therapy. Similar findings were observed in the present case study. However, unlike the previously reported cases, a retest of the blood culture was performed in the present case study, wherein the first blood culture was positive. Still, the second culture obtained during antibiotic therapy was negative. These findings indicate that a confirmed negative culture may not prevent the dilatation of an infected aneurysm (Table 2).

In patients with abdominal aortic aneurysms, the aortic wall's expression levels of proinflammatory cytokines (IL-1b, IL-6, IL-10, and TNF-a) increase. They are known to be even higher during sepsis [15]. This might explain the increase in the expression levels of the proinflammatory cytokines in the present case, where the CRP levels were not completely negative despite a negative blood culture test.

In light of the above findings, there was a high possibility that the infected aortic aneurysm dilated rapidly even if the antibiotic treatment was effective from an examination point of view. Furthermore, the infected fragile aortic wall dilated under continuous aortic pressure, regardless of the strict blood pressure control, and the dilating process could not be stopped. As the only way to treat such infected dilating aneurysms is surgical repair, inoperable patients need to be managed palliatively.

This case has several limitations: First, the pathogen of the pneumonia was not identified, not least because the sputum culture was not an adequate specimen at the time of the initial examination. Although there is evidence of distance between the site of pneumonia and the aorta, bloodstream infection from the shunt puncture site cannot be ruled out because sputum cultures were not obtained. If the sputum culture and blood culture matched, there would be further evidence that pneumonia is associated with an infected aneurysm. However, this may be difficult in practice as it has been reported that sputum cultures can be collected in about 60% of cases, of which only about 30% are considered adequate samples [16]. Second, pleural fluid culture could not be submitted due to lack of pleurodesis. If a pleural fluid culture could have been submitted, it would likely have further corroborated the invasion from the pneumonia into the pleural fluid and aorta. Third, cultures were not taken again on day 22 of admission when the inflammatory response and back pain worsened, and the choice of antimicrobial agent was not reconsidered. If the blood cultures had been tested again, the possibility of bacteria regrowth in the false lumen of the aneurysm or other bacteria establishment could have been further explored.

## Conclusions

To our knowledge, this is the first report on the dilation of an infected aneurysm caused by *S. aureus* despite clearance. Patients at high risk for infection and with calcifications in the blood vessels may develop infected aneurysms, which should not be overlooked, with the possibility of a rapid dilation. Early surgical treatment may lead to better results in operable patients, even if the results of blood tests and culture tests show an improving trend.
